# Trajectories of physical function and biological aging in generally healthy older adults with and without incident invasive cancer over a three-year follow-up: findings from the DO-HEALTH study

**DOI:** 10.1038/s41514-026-00360-2

**Published:** 2026-03-23

**Authors:** Wiebke Rösler, Melanie Kistler-Fischbacher, Stephanie Gängler, Markus G. Manz, Kressig W. Reto, John E. Orav, Bess Dawson-Hughes, Joanne Ryan, Daniel W. Belsky, Steve Horvath, Walter C. Willett, Maud Wieczorek, Heike A. Bischoff-Ferrari

**Affiliations:** 1 Department of Medical Oncology and Hematology, University Hospital Zurich, Zurich, Switzerland; 2 University of Basel, Department of Acute Aging Medicine FELIX PLATTER and Department of Geriatrics, University of Basel, Basel, Switzerland; 3 Swiss Campus for Healthy Longevity at the University of Basel, Basel, Switzerland; 4 Harvard Medical School, Boston, Massachusetts. Brigham and Women’s Hospital, Department of Medicine, Boston, MA USA; 5 Bone Metabolism Laboratory, Jean Mayer USDA Human Nutrition Research Center on Aging, Tufts University, Boston, MA USA; 6 Biological Neuropsychiatry & Dementia Unit, School of Public Health and Preventive Medicine, Monash University, Melbourne, Australia; 7 Department of Epidemiology, Butler Columbia Aging Center, Mailman School of Public Health, Columbia University, New York, NY USA; 8 Altos Labs, San Diego, CA USA; 9 Departments of Nutrition and Epidemiology, Harvard TH Chan School of Public Health, Harvard University, Boston, MA USA; 10 University of Zurich, Zurich, Switzerland

**Keywords:** Cancer, Health care, Medical research, Oncology

## Abstract

Cancer is associated with biological aging and functional decline; however, few studies have simultaneously examined objective changes in physical function and biological aging in older adults who develop cancer. We therefore compared functional and accelerated aging in generally healthy adults with and without incident invasive cancer, using data from DO-HEALTH, a three-year, randomized controlled trial including 2152 participants (mean age: 74.9 years, 61.1% women), free of major health conditions. Functional aging was assessed by the Short Physical Performance Battery, handgrip strength, gait speed, and five-times sit-to-stand test (STS). Biological aging was measured in a subsample (*n* = 777) using Horvath, Hannum, GrimAge and PhenoAge clocks, and DunedinPACE. Participants with incident cancer showed a greater decline in STS (Δ adjusted means [AM]: 0.64 s [95% CI 0.06, 1.22]) and grip strength (ΔAM −1.77 kPa [−3.51, −0.03]). Furthermore, baseline biological aging was accelerated between 3.52 and 6.77 months, measured by Horvath, Hannum, and PhenoAge clocks. Findings support the geroscience hypothesis linking greater phenotypic functional decline to accelerated biological aging in older adults with incident cancer. The data for this observational analysis stem from the DO-HEALTH clinical trial, which was prospectively registered (clinicaltrials.gov; NCT01745263; registered on December 10, 2012).

## Introduction

Cancer incidence increases exponentially with age^1^. Recent projections estimate that by 2035, approximately 60% of all incident cancers will occur in adults aged 65 years and older^1^. Therefore, cancer is considered a leading age-related disease^2^ often associated with multimorbidity and an increased risk of functional decline^3,4^.

Mechanistically, the decline in physical function – here defined as objective measures of physical performance such as gait speed or handgrip strength – in cancer patients may be caused by reduced physical activity, malnutrition, loss of appetite, and cachexia, which can lead to loss of skeletal muscle mass and thus function^3,5^. Other factors include fatigue, treatment toxicity, inflammation and other adverse effects that can affect physical function, such as polyneuropathy^6–8^. Cross-sectional studies have shown an association between a cancer diagnosis and decreased physical function among older adults^9–11^. Longitudinal studies suggest a similar association; however, the majority of evidence is limited by using self-reported data on physical function (e.g., questionnaires) and a lack of objective physical function measures, which is a critical shortcoming of the published literature^11–20^. Furthermore, many studies focused on a specific type of cancer (e.g., breast, lung)^14–17,20–22^, lack a non-cancer control^14,23^, or have a short follow-up (<3 years)^12,14,20^. Thus, longitudinal studies using objective measures of physical function and including non-cancer controls are warranted.

Aging and the pathogenesis of cancer share some biological mechanisms, one of which is the change in DNA methylation (DNAm). DNAm is a biomarker of biological aging and can be used to calculate epigenetic age acceleration (EAA), defined as the offset between chronological and biological age^24^. Previously, accelerated biological aging has been associated with an increased risk of incident cancer^25,26^, while cancer survival and cancer treatment have also been linked to accelerated aging^27,28^. In addition, different DNAm-based age measures have been developed that predict age itself, as well as different domains of aging or death. These measures may be differentially associated with systemic diseases such as cancer^29^. Yet, it is not known whether accelerated biological aging precedes cancer diagnosis or becomes evident only after diagnosis.

Most available studies have examined physical function domains separately or in parallel cohorts. For example, the Finnish Twin Study on Aging assessed associations between epigenetic clocks and physical function decline over three years in older women, but did not include incident cancer cases for comparison with non-cancer controls^30^. The Sister Study evaluated changes in DNA methylation-based aging metrics before and after breast cancer diagnosis and treatment, but did not simultaneously assess objective physical function measures in both cancer and non-cancer groups over the same interval^31^. To the best of our knowledge, longitudinal data that integrate biological aging and physical function within the same cohort, particularly in comparisons of individuals with incident cancer versus non-cancer controls, are lacking.

This study therefore had two aims. First, to compare three-year trajectories of objectively measured physical function between older adults who did and did not develop incident invasive cancer. Second, in a subset of participants with DNAm data, to compare baseline and three-year changes in biological age acceleration in those who did and did not develop incident invasive cancer. For this purpose, we used data from the DO-HEALTH trial, a large randomized controlled trial of 2,157 generally healthy, community-dwelling adults 70 years and older.

## Results

### Participant characteristics

Of the 2157 DO-HEALTH participants, 2125 had available baseline data and at least one follow-up measure for the four functional outcomes and were included in the present analysis. Table 1 shows the baseline characteristics of participants. Of the 2125 participants, 80 (3.8%) developed incident invasive cancer, whereas the remaining 2045 participants remained cancer-free during the three-year follow-up. If participants with a history of cancer five years prior to enrolment (*n* = 212) were excluded (sensitivity analysis), 69 participants had incident cancer and 1844 remained cancer-free. The mean age of participants was 74.9 years (standard deviation 4.3), 61.1% were women, and 83% were physically active at baseline.

**Table 1:** Baseline characteristics of the study population

	Overall (*n* = 2125)	Cancer free (*n* = 2045)	Cancer case (*n* = 80)	*p* value
Age [yrs], mean (SD)	74.89 (4.43)	74.86 (4.40)	75.71 (5.22)	0.092
Women, *n* (%)	1310 (61.1)	1272 (62.2)	38 (47.5)	0.011
BMI [kg/m^2^], mean (SD)	26.31 (4.29)	26.29 (4.29)	26.76 (4.29)	0.340
Prior fall, *n* (%)	884 (41.6)	846 (41.4)	38 (47.5)	0.329
Fried frailty status, *n* (%)				0.537
Robust	1133 (54.0)	1086 (53.8)	47 (58.8)	
Pre-frail	908 (43.3)	876 (43.4)	32 (40.0)	
Frail	5.8 (2.8)	57 (2.8)	1 (1.2)	
Physical activity frequency, *n* (%)				0.199
Inactive	363 (17.1)	351 (17.1)	11 (13.8)	
1–2 times/week	646 (30.3)	624 (30.3)	19 (23.8)	
≥3 times/week	1118 (52.7)	1118 (52.7)	50 (62.5)	
EQ-VAS score, mean (SD)^a^	0.90 (0.14)	0.90 (0.14)	0.91 (0.11)	0.520
MedDiet score, mean (SD)^b^	37.36 (5.03)	37.29 (5.05)	36.45 (4.51)	0.144
Polypharmacy, *n* (%)^c^	568 (26.7)	552 (27.0)	16 (20.0)	0.209
Gait speed [m/s], mean (SD)	1.12 (0.23)	1.12 (0.23)	1.14 (0.22)	0.359
SPPB score [0–12], median [IQR]	11.0 [10.0, 12.0]	11.0 [10.0, 12.0]	11.5 [11.0, 12.0]	0.434
STS [sec], mean (SD)	11.69 (4.27)	11.70 (4.28)	11.27 (4.13)	0.378
Handgrip strength, dominant hand [kPa], mean (SD)	60.33 (18.49)	60.18 (18.52)	64.21 (17.30)	0.056
History of cancer, *n* (%)^d^	212 (10.0)	201 (9.8)	11 (13.8)	0.338

Median [IQR], Man-Whitney U test.

*BMI* body mass index, *EQ-VAS* EuroQol Visual Analogue Scale, *IQR* interquartile range, *SD* standard deviation, *SPPB* Short Physical Performance Battery, *STS* five times sit-to-stand test.

^a^The EQ-VAS score ranges from 0 to 100 and higher scores are better.

^b^The MedDiet score ranges from 0 to 55 and higher scores indicate greater adherence to a Mediterranean Diet.

^c^Defined as taking ≥ five medications (both prescriptions and over-the-counter medications) taken regularly. Dietary supplements and alternative medicines were not considered.

^d^more than five years before the trial.

Among participants who developed invasive cancer, there was a smaller proportion of women (47.5 vs. 62.2%, *p* = 0.011) and handgrip strength was somewhat higher (64.2 vs. 60.2 kPa, *p* = 0.056). There were no other differences in participant characteristics at baseline.

Baseline characteristics of the subsample of 777 participants with available DNAm measures are presented in Supplementary Table 1. Of the 777 participants, 30 developed incident invasive cancer. Baseline characteristics did not differ between participants who developed cancer and those who stayed cancer-free, except that a higher proportion of participants who developed cancer had a history of cancer five years prior to enrolment (20.0% vs. 7.5%, *p* = 0.033). A comparison of Swiss participants with DNAm data (*n* = 777) and those without (*n* = 229) showed that individuals without DNAm data were slightly older (75.9 vs. 74.9 years) and had fewer years of education (12.9 vs. 13.5 years), but were otherwise comparable across key health and demographic characteristics. A full comparison is available in Bischoff-Ferrari et al., 2025^32^.

### Physical function

**Fig. 1:**
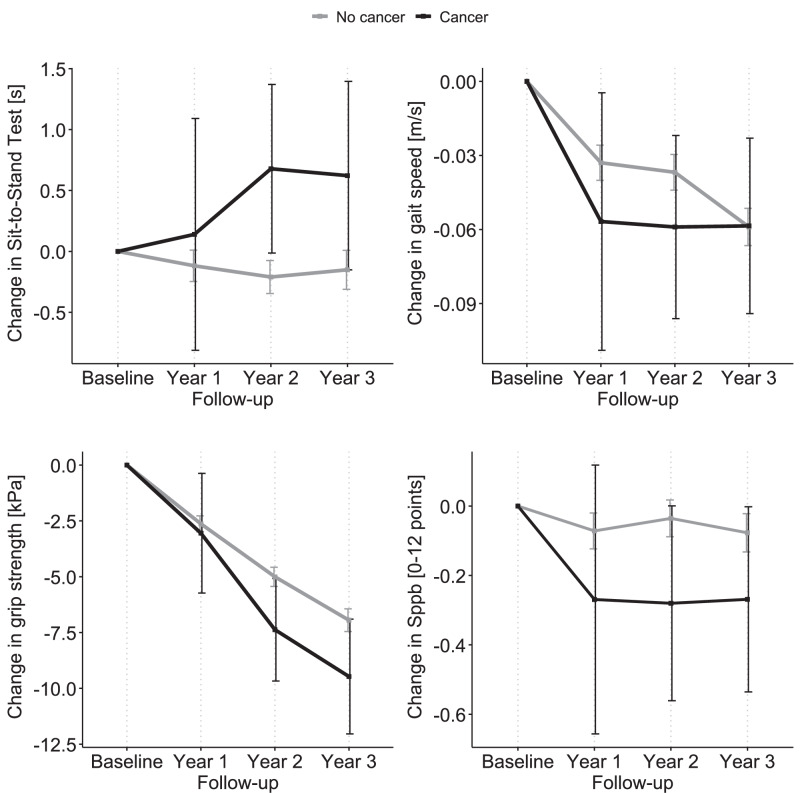
Changes from baseline in functional outcomes comparing individuals with or without incident invasive cancer over the three-year follow-up. Adjusted means of the change in functional outcomes from baseline over the three-year follow-up. The estimated differences in adjusted mean changes at each follow-up year are provided in Supplemental Table 8. Analyses were adjusted for DO-HEALTH treatments, age (continuous and categorical >85 years), sex, prior fall, BMI, study site, baseline level of the function measure and cancer history. *N* = 2125.

Over the three-year follow-up, participants with incident invasive cancer experienced a greater decline in STS performance compared to those without incident invasive cancer (difference in adjusted means [ΔAM]: 0.64 s, 95% CI 0.06 to 1.22, *p* = 0.032). Similarly, participants with incident invasive cancer compared to those without incident invasive cancer experienced a greater decline in grip strength (ΔAM: −1.77 kPa, 95% CI −3.51 to −0.03, *p* = 0.046). There were no significant differences for gait speed and SPPB, although the pattern was consistent for SPPB (Fig. 1, Supplementary Table 2). Adjusted mean changes at each year of follow-up are provided in Supplementary Table 3. In the sensitivity analysis excluding participants with a cancer history more than five years before enrollment, the association with STS remained significant (ΔAM: −1.70 kPa, 95% CI −3.57 to 0.17, *p* = 0.075), while the association with grip strength was no longer significant (ΔAM: 0.75 s, 95% CI 0.13 to 1.36, *p* = 0.018; Supplementary Table 2). There was a significant three-way interaction between incident cancer, time and sex for gait speed (*p* = 0.0125) and STS (*p* = 0.0137; Supplementary Table 4), therefore, we conducted a subgroup analysis. Female participants with incident cancer compared to female participants who remained cancer free, showed a greater decline in gait speed (ΔAM: −0.05 m/s, 95% CI −0.09 to −0.01) and STS (ΔAM: 1.11 s, 95% CI: 0.22 to 2.0; Supplementary Table 4).

### Biological aging

At baseline, participants who developed incident cancer over the three-year follow-up had accelerated biological aging (epigenetic age acceleration) measured by Horvath (ΔAM: 0.41, 95% CI 0.04 to 0.77, 3.52 months), Hannum (ΔAM: 0.52 95% CI 0.16 to 0.87, 4.98 months) and PhenoAge (ΔAM: 0.51, 95% CI 0.17 to 0.85, 6.77 months) clocks compared to those without incident cancer (Table 2). This remained consistent in the sensitivity analysis, adjusting for cell counts or additional confounders (Supplementary Table 5).

**Table 2:** Accelerated biological aging (Epigenetic Age Acceleration (EAA)) in participants with and without incident cancer at baseline

		Adjusted means (95% CI)		
Biological age measure	Mean (SD)	Cancer case (*n* = 30)	Cancer free (*n* = 747)	Difference (95% CI)
Horvath (PC adjusted)	−0.00 (0.73)	0.39 (0.03, 0.75)	−0.02 (−0.1, 0.06)	**0**.**41 (0**.**04, 0**.**77)**
Hannum (PC adjusted)	0.02 (0.76)	0.52 (0.17, 0.87)	0 (−0.08, 0.08)	**0**.**52 (0**.**16, 0**.**87)**
PhenoAge (PC adjusted)	0.08 (0.95)	0.49 (0.15, 0.83)	−0.02 (−0.1, 0.05)	**0**.**51 (0**.**17, 0**.**85)**
GrimAge (PC adjusted)	0.05 (1.02)	0.15 (−0.02, 0.32)	0 (−0.04, 0.04)	0.15 (−0.02, 0.32)
GrimAge2	0.00 (3.17)	0.28 (−0.05, 0.62)	0 (−0.07, 0.08)	0.28 (−0.06, 0.62)
DunedinPACE	0.97 (0.1)	0.29 (−0.05, 0.62)	0 (−0.07, 0.08)	0.28 (−0.05, 0.62)

*Adjustments: DO-HEALTH treatments, age (continuous and categorical* *>* 85 years), sex, prior fall, BMI, study site, cancer history. EAA is shown in standardized units. To calculate the effect in months: (Adjusted mean difference* SD+ mean) * 12.

In the analysis of GrimAge DNAm-based estimates of plasma proteins, only baseline beta-2 microglobulin (B2M) was higher in participants who developed incident cancer compared to those without incident cancer (ΔAM: 0.51, 95% CI 0.15 to 0.87; Supplementary Table 6). After adjusting for cell counts, the association for PC-DNAm B2M remained stable (Supplementary Table 4).

**Fig. 2:**
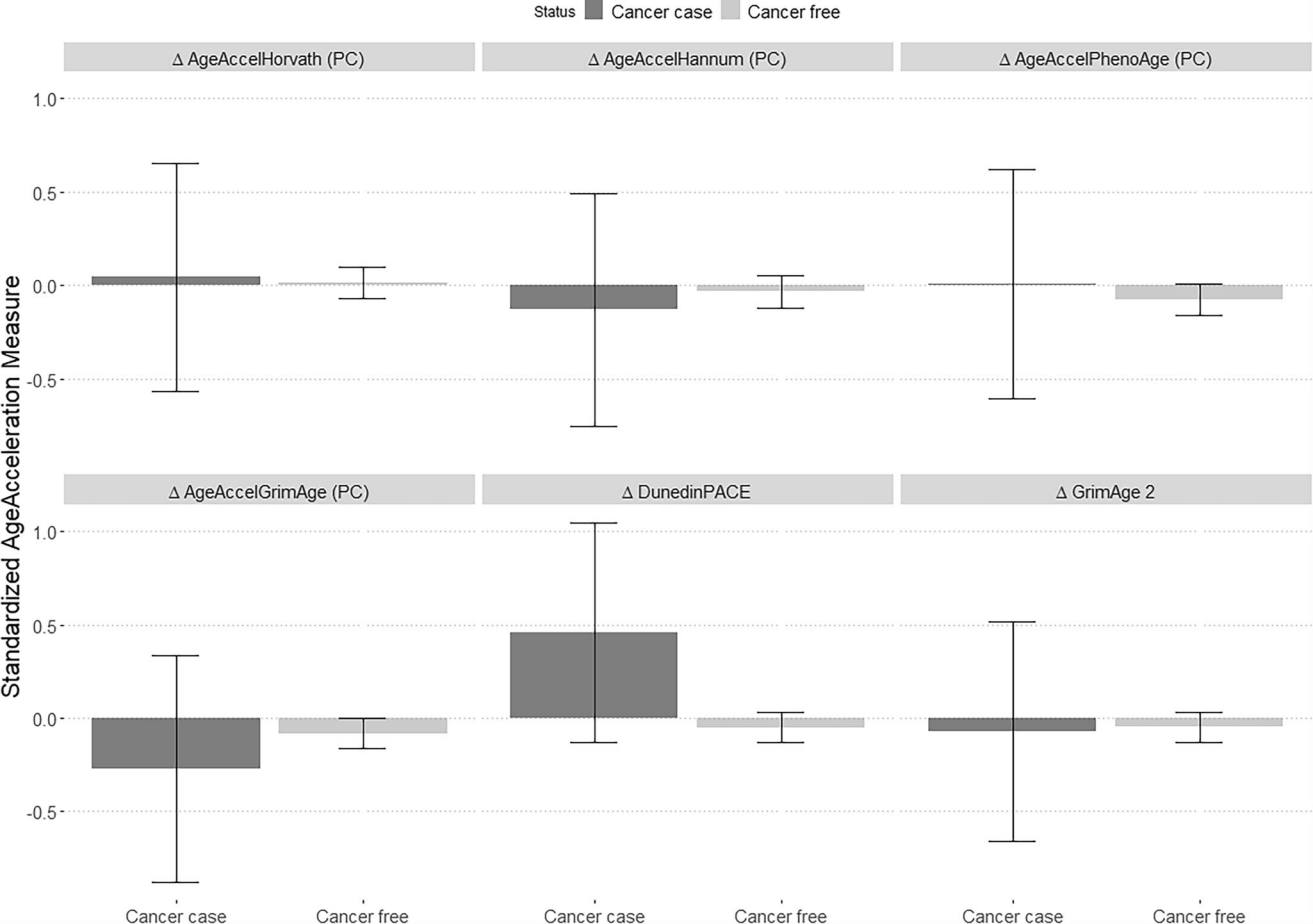
Bar plot of adjusted marginal means and 95% confidence intervals from generalized linear regression models showing the three-year changes in DNAm measures from baseline in participants with and without incident cancer. Adjusted for DO-HEALTH treatments, age (continuous and categorical > 85 years), sex, prior fall, BMI, study site and cancer history and time without cancer. *N* = 777.

Regarding the change in accelerated biological aging from baseline to year three, we did not document any differences between participants who developed incident cancer versus those who did not (Fig. 2, Supplementary Table 7). Adjusting this analysis for cell counts at baseline and their change resulted in an increase in PhenoAge in participants with incident cancer versus participants who remained cancer-free over the follow-up. Further, participants with incident cancer showed an increase in the DunedinPACE of aging from baseline to year three after the cell count adjustment (ΔAM 0.69, 95%CI 0.13 to 1.26; Supplementary Table 8). In the sensitivity analysis adjusted for cell counts, the change in the DNAm-based plasma protein increased for B2M, Cystatin C, and GDF15 in participants with incident cancer versus cancer-free participants (Supplementary Table 8). Additional adjustment for confounders did not change the absence of differences between participants without cancer and those who developed incident cancer (Supplementary Table 8).

## Discussion

Among the whole study sample of 2125 DO-HEALTH participants, we observed a greater decline in objectively measured physical function (STS, grip strength) among participants who developed incident invasive cancer compared to those who did not over a three-year follow-up. Furthermore, supporting the geoscience theory, we observed accelerated baseline biological aging in participants who were diagnosed with incident invasive cancer during the three-year follow-up. This was in a subset of 777 Swiss DO-HEALTH participants, who had DNAm data available.

Our observation that participants with incident invasive cancer experience a greater decline in objectively measured physical function is consistent with a previous study, which reports significantly greater declines in grip strength and gait speed in breast cancer survivors aged 65 and older compared to cancer-free controls over a 20-year follow-up^22^. In contrast, two other studies did not report any differences in physical function decline (e.g., grip strength, gait speed, quadriceps strength) after cancer diagnosis, compared to control, and among generally healthy, community-dwelling older adults (70–79 years) with different types of incident cancer after a short median follow-up of six months^33^, and among men (mean age 76 years) with prostate cancer^21^. Beyond the few studies using objective tests of physical function, most studies have relied on self-reported measures, which likewise report greater decline in physical function in individuals developing cancer compared to controls^12,15–17,19^. However, self-reported measures of physical function are prone to overestimating – or less commonly, underestimating – functional capabilities and they do not quantify nor distinguish the different components that contribute to daily physical function, such as strength, mobility or balance^34^. The lack of significance for the SPPB, despite a clear effect for STS, may be explained by the inclusion of balance and gait speed in the composite score. Balance was not analyzed separately in our study, and gait speed did not differ significantly between groups. While the reason for the null finding in gait speed is unclear, it may reflect differences in the sensitivity of the individual. To the best of our knowledge, and therefore contributing to the literature, our study is unique for its repeated assessment of physical function based on standardized objective tests.

Our subgroup findings for participants in DO-HEALTH with DNAm data suggest that individuals who develop incident invasive cancer over three years have accelerated biological aging already at baseline prior to their new cancer diagnosis. This is supported by several studies showing an association between DNA methylation-based estimates of biological age and risk of incident cancer^25,26^. For example, a pooled analysis of prospective studies including 41,513 healthy adults (99% aged between 40–69 years) found a 20% increased cancer risk for the highest versus the lowest quartile of EAA measured by Horvath and Hannum epigenetic clocks^25^. Similarly, a 15-30% increased cancer risk for the fourth versus first quartile of EAA measured by PhenoAge and GrimAge was documented in a smaller sample of 4942 participants^35^. Further, changes in methylation patterns that could contribute to cancer have been identified in aging tissues, pre-malignant lesions and tumours^26^. Examples include hypermethylation and thus silencing of tumour suppressor gene promoters, mismatch repair genes and polycomb group proteins, which are required for stem cell differentiation^26^.

Notably, our data does not support a further increase in EAA from baseline to year three in participants who developed incident invasive cancer versus those who did not. These findings support a metrics of aging where changes in biological aging happen prior to phenotypic aging of disease incidence and functional decline^36^. Additionally, our data suggests that epigenetic aging may play a role in tumorigenesis^26,37^. More work is needed to investigate epigenetic alterations as well as other accompanying omics changes that may drive or accompany cancer development^26^.

Our study has several strengths. First, it is based on a large sample of 2125 generally healthy adults aged 70 years and older, followed longitudinally with repeated, standardized, objective measures of physical function. Second, all participants were cancer-free in the five years prior to enrolment, which reduces bias from recent cancer diagnosis. To best account for cancer latency, we performed a sensitivity analysis excluding individuals with a cancer history prior to these five years, which yielded consistent findings. Third, this study extends phenotypic functional aging among individuals with incident invasive cancer to accelerated biological aging prior to their diagnosis of incident invasive cancer and functional decline, which is in support of the geroscience theory^36^. Finally, all cancer cases were confirmed by medical records, and functional tests were performed with standardized protocols by certified DO-HEALTH study staff across all centers.

Our study also has limitations. First, while we have functional measures in all DO-HEALTH participants, DNAm data at baseline and year three were only available in the Swiss participants. Second, we acknowledge the importance of lifestyle factors for both biological aging and cancer incidence. We could not adjust for all lifestyle factors in the main models due to the number of design variables. However, we conducted a sensitivity analysis including smoking status as a covariate, and we included diet and physical activity in the baseline characteristics. Yet, residual confounding cannot be excluded and is inherent to the study design. Third, because functional assessments were scheduled within a narrow window around annual visits and cancer diagnosis was aligned to planned time points, some degree of time misclassification cannot be excluded, although we expect any resulting bias to be minimal. Fourth, we acknowledge that the large number of statistical tests performed may increase the risk of chance findings, and this should be considered when interpreting our results. Fifth, DO-HEALTH only includes European participants from five countries (Switzerland, Germany, Austria, France, Portugal) and results may not be generalizable to other populations, particularly as race may affect epigenetic aging^27^. Finally, DO-HEALTH participants were relatively healthy without major comorbidities. Thus, our findings may not extend to more vulnerable older adults.

In conclusion, individuals aged 70 years and older who were generally healthy at the onset of our study and developed incident invasive cancer within three years showed a greater decline in physical function over time compared to individuals who were not diagnosed with incident invasive cancer. Additionally, prior to their diagnosis of incident invasive cancer and functional decline, these individuals had, on average, accelerated biological aging. Further studies are needed to define the potential use of biomarkers of biological aging in early cancer diagnosis and health promotion.

## Methods

### Study design and participants

This is an observational, longitudinal analysis using data from the DO-HEALTH trial. DO-HEALTH was a three-year, multi-center, double-blinded, randomized controlled trial in generally healthy older adults ( ≥ 70 years). The full study protocol has been published^38^, as have the effects of the interventions on the primary outcomes^39^ and on incident invasive cancer^40^. In brief, 2,157 community-dwelling adults were recruited from seven centers in five European countries (Switzerland, Germany, Austria, France, and Portugal). The main inclusion criteria were absence of major health events in the five years prior to enrollment (including new or recurrent cancer diagnosis, treatment), sufficient mobility to come to the study center, and good cognitive function (Mini Mental State Examination [MMSE] score ≥24). For the present analysis, we only included participants for whom data for all four functional tests at baseline were available.

The trial protocol was registered (clinicaltrials.gov; NCT01745263; registered December 10, 2012) and approved by regulatory agencies of all five countries. Approval for this observational analysis was obtained after trial completion from the ethics committee in Zurich (Reference number: Nr. 2024-00935). All procedures were conducted in accordance with the Declaration of Helsinki. For the analyses on accelerated biological aging, DNAm measures were available in the subgroup of 777 Swiss participants at baseline.

### Incident cancer

Cancer events were assessed prospectively every three months across the three-year follow-up (yearly clinical visits and three-monthly phone calls between visits)^40^. If a new cancer case was reported, a detailed report was completed and medical records were reviewed in detail to verify each new cancer event by an Independent Physician Endpoint Committee. All types of cancers were considered, except for non-melanoma skin cancer and benign neoplasms. An incident cancer case was assigned to the closest timepoint of its incidence. For example, if a case occurred two months after the year one visit, its incidence was assigned to the visit at year one and from this time point on coded as “1” in the regression.

### Physical function

Standardized physical function measures were assessed in the entire DO-HEALTH population at baseline, year one, two and three. These included the Short Physical Performance Battery (SPPB), gait speed, five times sit-to-stand test (STS) and grip strength^38^. The SPPB is a standardized test of lower extremity function, comprising balance, gait speed, and STS components, with a maximum score of 12 points indicating best performance^38^. The outcomes gait speed and STS, were measured as components of the SPPB. Specifically, the time in seconds to complete the STS test was recorded from the initial seated position to completion of the fifth stand. Gait speed (m/s) was assessed over a 4-metre distance. Handgrip strength was determined using a Martin Vigorimeter (KLS Martin KG, Tuttlingen, Germany) and the best of three consecutive attempts was used^41^.

### DNAm data and epigenetic clocks

DNA methylation data were available for a subsample of 777 participants from Switzerland who provided consent for further use of biological material, had blood samples available at both baseline and year three, and whose samples met array quality-control criteria. Whole blood samples were collected in PAXgene DNA tubes. Blood aliquots were sent to Life&Brain (Department of Genomics, Bonn, Germany) for DNA extraction. The Infinium Methylation EPIC Bead Array version 1 (Illumina, Inc.: “EPIC array”) was then used to generate genome-wide methylation profiling. Briefly, the array targets more than 850,000 CpG sites in the human genome, quantifying 5-methylcytosine levels at each interrogated locus. Beta values were extracted and used for the analysis. The EPIC arrays were processed in accordance with the manufacturer’s instructions and scanned using the Illumina iScan platform. Baseline and 36-month samples from each individual were processed in the same array batch and on the same BeadChip to reduce batch effects. For quality control and normalization analyses, the minfi (v.1.42.0) Bioconductor (v.2.46.0) R statistical programming packages (v.3.6.3) were used^42^. Probes with detection *P* values > 0.01 were considered missing in a sample and if they were missing in >50% of sample, they were excluded from the analysis. The minfi default methods were used for normalization and elimination of systematic dye bias in two-channel probes.

The sample results were then used to calculate six distinct biological aging estimations: For Horvath, Hannum and PhenoAge clocks, and GrimAge versions constructed from DNAm principal components (PCs) were analyzed using the Higgins-Chen method, as they have superior technical reliability compared to original versions^24,43–45^. GrimAge2 and DunedinPACE demonstrate strong reliability in their original versions^46^. To compute GrimAge2 and the DNAm-based protein estimates included in the GrimAge clock, selected CpGs were submitted to the DNA methylation clock calculator hosted by the Horvath Lab (https://dnamage.genetics.ucla.edu/home; 19.03.2024) to derive the DNAm clock estimates and DNAm protein estimates. PC versions of the GrimAge, PhenoAge, Horvath, and Hannum epigenetic clocks were computed for the same samples as the GrimAge DNAm proteins according to the method described by Higgins-Chen et al. using the R code hosted on GitHub (https://github.com/MorganLevineLab/PC-Clocks) using R (version 4.2.1)^45^. DunedinPACE was calculated for the same samples as the other DNAm variables according to the method described by Belsky et al. using the R code hosted on GitHub (https://github.com/danbelsky/DunedinPACE/) using R (version 4.2.1)^46^. After processing by the clock calculator, samples were excluded if there was a mismatch between reported sex and predicted biological sex by the clock calculator (*n* = 3) and those who had a low-quality DNA extraction as indicated by the lab (*n* = 6). Following quality control and normalization, DNAm data at baseline and 36 months were available for 777 Swiss participants.

Epigenetic age acceleration (EAA) was derived from the residuals of the regression of each respective clock measure on chronological age.

### Additional baseline variables

To provide a more comprehensive description of the study population, several additional variables are reported in the baseline characteristics. Self-rated health was assessed with the EuroQol Visual Analogue Scale (EQ-VAS), where participants rated their overall health on a 0–100 scale, with higher scores indicating better health^47^. Frailty phenotype was defined according to the Fried frailty phenotype criteria and based on weight loss, exhaustion, low activity, slowness, and weakness^48^. Physical activity was self-reported using an excerpt from the Nurses’ Health Study physical activity questionnaire, capturing time spent with leisure-time physical activity, walking or standing^49^. Dietary intake was assessed with a 216-item food frequency questionnaire (FFQ) developed for older adults, from which we calculated adherence to a Mediterranean dietary pattern using the MedDietScore^50^. Medication use was assessed through participant self-report and medication lists and polypharmacy was defined as the use of ≥ 5 medications^51^.

### Statistical analysis

Variables were assessed for their distribution using histograms and quantile plots. Normally distributed variables were presented as mean and standard deviation and non-normally distributed data as median and interquartile range. Categorical variables were presented as frequencies and percentages. Correlations between outcomes were assessed using Spearman's rank correlation.

Separate linear mixed effect models were used to compare changes from baseline in functional outcomes in participants who had a cancer diagnosis versus no cancer diagnosis over the follow-up. Each participant had up to three outcome measures (changes in functional outcomes from baseline at one, two, and three years) and the primary predictors were cancer status, follow-up year, and the interactions between these two predictors. An unstructured covariance matrix was specified in the repeated statement. Parameters were estimated using restricted maximum likelihood. All analyses were adjusted for the DO-HEALTH stratification variables, including age (continuous and categorical >85years), sex, prior fall, BMI, and study site, and in addition for baseline level of the outcomes, history of cancer (>5 years prior enrollment) and the DO-HEALTH treatments. Cancer status was assessed as a time-varying covariate, depending on the cancer incidence over the three years and had an interaction with time. Adjusted mean differences in changes (MD) with 95% confidence intervals (CI) are presented. Two separate sensitivity analyses were conducted. First, to assess robustness of the findings to prior cancer history, we excluded all participants with a history of cancer more than five years before study start (*n* = 217). Second, to evaluate the influence of additional potential confounders, we repeated the analysis in the full cohort while additionally adjusting the models for the number of comorbidities, smoking status, and years of education. Subgroup analyses by biological sex were performed when there was evidence for effect modification (*P* < 0.05) in the interaction between cancer status, time and sex.

The associations of baseline DNAm measures and of three-year changes in biological age acceleration with incident invasive cancer status were assessed in separate generalized linear regression models, adjusted for DO-HEALTH treatments, age (continuous and categorical >85 years), sex, prior fall, BMI, study site and cancer history (>5 years prior to enrollment). The models for three-year change were additionally adjusted for DO-HEALTH treatment groups, baseline biological age acceleration, and days to cancer (for participants with cancer; participants without cancer were assigned a value of zero). Model parameters were estimated using ordinary least squares, and normality of the error term was evaluated with quantile plots and histograms. To test the influence of cell composition of the samples, a sensitivity analysis adjusted for DNAm estimated cell counts (eosinophils, basophils, monocytes, natural killer cells, CD8 cytotoxic T cells, and CD4 cells) was performed for both baseline DNAm and change in DNAm from baseline^52^. To test the effect of lifestyle factors and socioeconomic status, a sensitivity analysis adjusted for current smoking status, comorbidities and years of education at baseline was done.

All analyses were conducted using SAS (version 9.4) and R (version 4.2) in R Studio. Significance threshold was set to *P* < 0.05.

## Supplementary information

DO-HEALTH_cancer_function EAA_supplemental file_Revised_2025-11-03

## Data Availability

Data from DO-HEALTH, used in the context of this project, as well as codebooks and analytic codes, will initially be reserved for the primary researchers of the Center of Aging and Mobility Research Group to fully exploit the datasets. However, all data supporting the findings of this study are available from the corresponding author upon request.

## Code availability

Previously published code was used in this study and no new code was generated^45,46^.
