# Supplementary material for: Trajectories of physical function and biological aging in generally healthy older adults with and without incident invasive cancer over a three-year follow-up: findings from the DO-HEALTH study
Source: NPJ Aging. 2026 Mar 23;12(1):99. doi: 10.1038/s41514-026-00360-2 (PMC13396198; doi:10.1038/s41514-026-00360-2)
Supplement: Supplementary file 1 — DO-HEALTH_cancer_function EAA_supplemental file_Revised_2025-11-03 [file 41514_2026_360_MOESM1_ESM.docx]

## **SUPPLEMENTARY MATERIAL**

## **Supplementary Table 1**. **Baseline characteristics of the Swiss study population with available DNAm measures**

|  | **Overall**  (n = 777) | **Cancer free**  (n = 747) | **Cancer case**  (n = 30) | ***P* value** |
| --- | --- | --- | --- | --- |
| Age [yrs], mean (SD) | 74.98 (4.45) | 74.96 (4.45) | 75.50 (4.62) | 0.513 |
| Women, n (%) | 464 (59.7) | 450 (60.2) | 14 (46.7) | 0.195 |
| BMI [kg/m^2^], mean (SD) | 25.72 (4.04) | 25.67 (4.00) | 26.92 (4.89) | 0.097 |
| Prio fall, m(%) | 320 (41.2) | 308 (41.2) | 12 (40) | 1.000 |
| Fried frailty status, n (%) |  |  |  | 0.747 |
| Robust | 450 (58.3) | 431 (58.1) | 19 (63.3) |  |
| Pre-frail | 314 (40.7) | 303 (40.8) | 11 (36.7) |  |
| Frail | 8 (1.0) | 8 (1.1) | 0 (0.0) |  |
| Physical activity frequency, n (%) |  |  |  | 0.939 |
| Inactive | 93 (12.0) | 89 (11.9) | 4 (13.3) |  |
| 1-2 times/week | 227 (29.2) | 219 (29.3) | 8 (23.7) |  |
| > 3 times/week | 457 (58.8) | 439 (58.8) | 18 (60.0) |  |
| EQ-VAS score, mean (SD) ^a^ | 0.93 (0.11) | 0.93 (0.11) | 0.91 (0.09) | 0.296 |
| MedDiet score, mean (SD) ^b^ | 37.62 (4.75) | 37.60 (4.79) | 38.13 (3.43) | 0.544 |
| Polypharmacy, n (%) ^c^ | 143 (18.4) | 138 (18.5) | 5 (16.7) | 0.992 |
| Gait speed [m/s], mean (SD) | 1.11 (0.21) | 1.11 (0.21) | 1.09 (0.19) | 0.488 |
| SPPB score [0-12], median [IQR] | 12.00 [11.00, 12.00] | 12.00 [11.00, 12.00] | 12.00 [11.00, 12.00] | 0.833 |
| STS [sec], mean (SD) | 10.66 (2.89) | 10.64 (2.81) | 11.30 (4.50) | 0.223 |
| Grip strength, dominant hand [kPA], mean (SD) | 62.33 (18.44) | 62.23 (18.50) | 64.93 (17.01) | 0.431 |
| History of cancer, n (%) ^d^ | 62 (8.0) | 56 (7.5) | 6 (20.0) | 0.033 |
| Median [IQR], Man-Whitney U test  Abbreviations: BMI, body mass index; IQR, interquartile range; SD, standard deviation; SPPB, Short physical performance battery; STS, five times sit-to-stand test  ^a^ The EQ-VAS score ranges from 0 to 100 and higher scores are better  ^b^ The MedDiet score ranges from 0 to 55 and higher scores indicate greater adherence to a MedDiet  ^c^ Defined as taking ≥ five medications (both prescriptions and over-the-counter medication) taken regularly. Dietary supplements and alternative medicines were not considered.  ^d^ More than five years before enrollment in the trial | | | | |

## **Supplementary Table 2**. **Changes in functional outcomes from baseline by cancer status for the main analysis and the sensitivity analysis omitting participants with a history of cancer**

|  | **Adjusted means (95%CI)** | |  | |  |
| --- | --- | --- | --- | --- | --- |
| **Outcome** | **Cancer free** | **Cancer case** | | **Difference (95%CI)** | ***p* value** |
| **Main analysis (confirmed cancer cases)** |  |  | |  |  |
| ∆ STS-test [s] | -0.16 (-0.27, -0.05) | 0.48 (-0.1, 1.06) | | 0.64 (0.06, 1.22) | 0.032 |
| ∆ Gait speed [m/s] | -0.02 (-0.06, 0.03) | -0.06 (-0.44, 0.31) | | -0.04 (-0.42, 0.33) | 0.822 |
| ∆ Grip strength [kPa] | -4.86 (-5.19, -4.53) | -6.63 (-8.36, -4.91) | | -1.77 (-3.51, -0.03) | 0.046 |
| ∆ SPPB | -0.06 (-0.1, -0.02) | -0.27 (-0.49, -0.05) | | -0.21 (-0.43, 0.01) | 0.063 |
| **Sensitivity analysis (no history of cancer 5 years prior to enrolment)** |  |  | |  |  |
| ∆ STS-test [s] | -0.17 (-0.29, -0.05) | 0.58 (-0.03, 1.19) | | 0.75 (0.13, 1.36) | 0.018 |
| ∆ Gait speed [m/s] | -0.02 (-0.07, 0.03) | -0.07 (-0.5, 0.35) | | -0.06 (-0.49, 0.37) | 0.798 |
| ∆ Grip strength [kPa] | -4.83 (-5.18, -4.48) | -6.53 (-8.39, -4.68) | | -1.70 (-3.57, 0.17) | 0.075 |
| ∆ SPPB | -0.04 (-0.09, 0) | -0.25 (-0.48, -0.02) | | -0.21 (-0.44, 0.03) | 0.084 |
| **Sensitivity analysis (adjustment for number of comorbidities, smoking status, education years** |  |  | |  |  |
| ∆ STS-test [s] | -0.17 (-0.29, -0.06) | 0.41 (-0.16, 0.99) | | 0.58 (0, 1.17) | 0.049 |
| ∆ Gait speed [m/s] | -0.04 (-0.05, -0.04) | -0.05 (-0.08, -0.02) | | -0.01 (-0.04, 0.02) | 0.464 |
| ∆ Grip strength [kPa] | -4.84 (-5.17, -4.51) | -6.56 (-8.29, -4.84) | | -1.72 (-3.47, 0.02) | 0.053 |
| ∆ SPPB | -0.06 (-0.1, -0.01) | -0.24 (-0.46, -0.02) | | -0.18 (-0.4, 0.04) | 0.106 |
| Estimates are aggregated over all measured time points. Adjustments: age (continuous and categorical >85 years), sex, prior fall, BMI, study site, baseline level of the function measure, cancer history (main analysis), DO-HEALTH treatment | | | | | |

## **Supplementary Table 3. Adjusted mean changes (95% CI) in functional outcomes at each follow-up year for participants with and without incident invasive cancer, and the estimated between-group differences**

|  |  | **Adjusted Means (95% CI)** | | |  |
| --- | --- | --- | --- | --- | --- |
| **Outcome** | **Time point** | **Cancer free** | **Cancer case** | **Difference** | ***p* value** |
| ∆ STS-test [s] | Year 1 | -0.12 (-0.25, 0.01) | 0.14 (-0.81, 1.09) | 0.26 (-0.7, 1.21) | 0.597 |
|  | Year 2 | -0.21 (-0.35, -0.07) | 0.68 (-0.01, 1.37) | 0.89 (0.19, 1.59) | 0.013 |
|  | Year 3 | -0.15 (-0.31, 0.01) | 0.62 (-0.15, 1.4) | 0.77 (-0.01, 1.56) | 0.054 |
| ∆ Gait speed [m/s] | Year 1 | -0.03 (-0.04, -0.03) | -0.06 (-0.11, 0) | -0.02 (-0.08, 0.03) | 0.373 |
|  | Year 2 | -0.04 (-0.04, -0.03) | -0.06 (-0.1, -0.02) | -0.02 (-0.06, 0.02) | 0.247 |
|  | Year 3 | -0.06 (-0.07, -0.05) | -0.06 (-0.09, -0.02) | 0 (-0.04, 0.04) | 0.981 |
| ∆ Grip strength [kPa] | Year 1 | -2.63 (-2.99, -2.28) | -3.05 (-5.73, -0.37) | -0.42 (-3.11, 2.28) | 0.761 |
|  | Year 2 | -5 (-5.43, -4.57) | -7.37 (-9.67, -5.08) | -2.37 (-4.69, -0.05) | 0.046 |
|  | Year 3 | -6.95 (-7.46, -6.44) | -9.47 (-12.04, -6.9) | -2.52 (-5.14, 0.09) | 0.058 |
| ∆ SPPB | Year 1 | -0.07 (-0.12, -0.02) | -0.27 (-0.66, 0.12) | -0.2 (-0.59, 0.19) | 0.320 |
|  | Year 2 | -0.04 (-0.09, 0.02) | -0.28 (-0.56, 0) | -0.24 (-0.53, 0.04) | 0.091 |
|  | Year 3 | -0.08 (-0.13, -0.02) | -0.27 (-0.54, 0) | -0.19 (-0.46, 0.08) | 0.165 |
| Abbreviations: SPPB, Short Physical Performance Battery; STS, five times sit-to-stand | | | | | |

## **Supplementary Table 4**. **Changes in functional outcomes from baseline by cancer status for subgroups by sex**

|  |  | **Adjusted means (95% CI)** | |  | |  |  |
| --- | --- | --- | --- | --- | --- | --- | --- |
| **Outcome** | **Subgroup** | **Cancer free** | **Cancer case** | | **Difference (95%CI)** | ***p* value** | **Interaction p-value ^a^** |
| ∆ Gait speed [m/s] | Male | -0.04 (-0.05, -0.03) | -0.02 (-0.06, 0.02) | | 0.02 (-0.02, 0.07) | 0.331 | 0.0125 |
|  | Female | -0.04 (-0.05, -0.03) | -0.09 (-0.13, -0.05) | | -0.05 (-0.09, -0.01) | 0.021 |  |
| ∆ STS-test [s] | Male | -0.23 (-0.39, -0.07) | 0.01 (-0.71, 0.73) | | 0.24 (-0.48, 0.97) | 0.510 | 0.0137 |
|  | Female | -0.12 (-0.27, 0.04) | 0.99 (0.11, 1.88) | | 1.11 (0.22, 2) | 0.015 |  |
| ^a^ cancer status* time*sex  Estimates presented are summed over all time points. Adjustments: age (continuous and categorical >85 years), prior fall, BMI, study site, baseline level of the function measure, cancer history (main analysis), DO-HEALTH treatment | | | | | | | |

**Supplementary Table 5. Sensitivity analysis of the baseline DNAm measures in participants with and without incident cancer adjusted for cell counts and adjusted for lifestyle factors (smoking status, education years, number of comorbidities)**

|  | **Adjusted means (95% CI)** | | | | |
| --- | --- | --- | --- | --- | --- |
| **Biological age measure** | **Cancer case (n = 30)** | | **Cancer free (n = 747)** | | **Difference** |
| **Sensitivity analysis adjusted for cell counts** |  | |  | |  |
| Horvath (PC adjusted) | 0.38 (0.04, 0.72) | | -0.02 (-0.1, 0.05) | | 0.4 (0.06, 0.75) |
| Hannum (PC adjusted) | 0.52 (0.2, 0.84) | | -0.01 (-0.09, 0.06) | | 0.53 (0.21, 0.86) |
| PhenoAge (PC adjusted) | 0.5 (0.2, 0.79) | | -0.03 (-0.1, 0.04) | | 0.53 (0.23, 0.83) |
| GrimAge (PC adjusted) | 0.14 (-0.02, 0.3) | | 0.01 (-0.03, 0.04) | | 0.13 (-0.03, 0.29) |
| GrimAge2 | 0.31 (-0.01, 0.63) | | 0 (-0.07, 0.07) | | 0.31 (-0.02, 0.63) |
| DunedinPACE | 0.29 (-0.03, 0.61) | | 0 (-0.07, 0.08) | | 0.29 (-0.03, 0.62) |
| **GrimAge DNAm based plasma proteins** | |  | |  | |
| PC-DNAm ADM | -0.02 (-0.07, 0.03) | | -0.01 (-0.02, 0) | | -0.01 (-0.06, 0.04) |
| PC-DNAm B2M | 0.49 (0.17, 0.81) | | 0.01 (-0.06, 0.08) | | 0.48 (0.15, 0.81) |
| PC-DNAm TIMP-1 | 0.03 (-0.1, 0.17) | | -0.01 (-0.04, 0.02) | | 0.05 (-0.09, 0.18) |
| PC-DNAm Leptin | -0.01 (-0.02, 0) | | 0 (0, 0) | | -0.01 (-0.02, 0) |
| PC-DNAm CystatinC | 0.28 (-0.02, 0.58) | | 0.01 (-0.06, 0.07) | | 0.27 (-0.03, 0.58) |
| PC-DNAm PAI-1 | -0.05 (-0.19, 0.09) | | -0.03 (-0.06, 0) | | -0.02 (-0.16, 0.12) |
| PC-DNAm GDF15 | 0.01 (-0.28, 0.3) | | -0.05 (-0.11, 0.02) | | 0.06 (-0.24, 0.36) |
| **Sensitivity analysis adjusted for smoking, education years and comorbidities** |  | |  | |  |
| Horvath (PC adjusted) | 0.4 (0.04, 0.75) | | -0.01 (-0.1, 0.07) | | 0.41 (0.04, 0.77) |
| Hannum (PC adjusted) | 0.5 (0.15, 0.85) | | 0 (-0.08, 0.07) | | 0.51 (0.15, 0.86) |
| PhenoAge (PC adjusted) | 0.46 (0.12, 0.79) | | -0.03 (-0.1, 0.05) | | 0.48 (0.14, 0.83) |
| GrimAge (PC adjusted) | 0.12 (-0.05, 0.28) | | -0.01 (-0.04, 0.03) | | 0.12 (-0.04, 0.29) |
| GrimAge2 | 0.22 (-0.11, 0.54) | | -0.01 (-0.09, 0.06) | | 0.23 (-0.1, 0.56) |
| DunedinPACE | 0.26 (-0.07, 0.59) | | 0 (-0.08, 0.07) | | 0.26 (-0.08, 0.6) |
| **GrimAge DNAm based plasma proteins** |  | |  | |  |
| PC-DNAm ADM | -0.03 (-0.09, 0.03) | | -0.01 (-0.03, 0) | | -0.02 (-0.08, 0.04) |
| PC-DNAm B2M | 0.53 (0.17, 0.88) | | 0.02 (-0.06, 0.1) | | 0.51 (0.15, 0.86) |
| PC-DNAm TIMP-1 | -0.01 (-0.22, 0.2) | | -0.02 (-0.07, 0.03) | | 0.01 (-0.2, 0.22) |
| PC-DNAm Leptin | -0.01 (-0.02, 0) | | 0 (0, 0) | | -0.01 (-0.02, 0) |
| PC-DNAm CystatinC | 0.24 (-0.09, 0.57) | | 0.01 (-0.07, 0.08) | | 0.23 (-0.1, 0.57) |
| PC-DNAm PAI-1 | -0.11 (-0.28, 0.06) | | -0.03 (-0.07, 0.01) | | -0.08 (-0.25, 0.1) |
| PC-DNAm GDF15 | -0.11 (-0.46, 0.23) | | -0.06 (-0.14, 0.02) | | -0.05 (-0.4, 0.3) |
| Adjustments: DO-HEALTH treatments, age (continuous and categorical >85 years), sex, prior fall, BMI, study site, cancer history, cell counts (NK, BAS, MONO, EOS, Bcells, CD4, CD8). EAA is shown in standardized units | | | | | |

## **Supplementary Table 6**. **DNAm-based proteins in participants with and without incident cancer at baseline**

|  | **Adjusted means (95% CI)** | | |
| --- | --- | --- | --- |
| **Biological age measure** | **Cancer case**  **(n = 30)** | **Cancer free**  **(n = 747)** | **Difference** |
| PC-DNAm ADM | -0.03 (-0.08, 0.03) | -0.01 (-0.02, 0) | -0.02 (-0.07, 0.04) |
| PC-DNAm B2M | 0.53 (0.17, 0.88) | 0.02 (-0.06, 0.1) | 0.51 (0.15, 0.87) |
| PC-DNAm TIMP-1 | 0.01 (-0.2, 0.22) | -0.02 (-0.06, 0.03) | 0.03 (-0.18, 0.24) |
| PC-DNAm Leptin | -0.01 (-0.02, 0) | 0 (0, 0) | -0.01 (-0.02, 0) |
| PC-DNAm CystatinC | 0.28 (-0.05, 0.61) | 0.02 (-0.06, 0.09) | 0.26 (-0.07, 0.6) |
| PC-DNAm PAI-1 | -0.09 (-0.26, 0.08) | -0.03 (-0.07, 0.01) | -0.06 (-0.23, 0.11) |
| PC-DNAm GDF15 | -0.04 (-0.39, 0.31) | -0.05 (-0.12, 0.03) | 0 (-0.35, 0.36) |
| Adjustments: DO-HEALTH treatments, age (continuous and categorical >85 years), sex, prior fall, BMI, study site, cancer history | | | |

## **Supplementary Table 7. Three-year changes in DNAm measures from baseline in participants with and without incident cancer**

|  | **Adjusted change from BL (95% CI)** | | |
| --- | --- | --- | --- |
| **Biological age measure** | **Cancer case (n = 30)** | **Cancer free (n =747)** | **Difference** |
| Horvath (PC adjusted) | 0.04 (-0.56, 0.65) | 0.02 (-0.07, 0.1) | 0.03 (-0.6, 0.66) |
| Hannum (PC adjusted) | -0.13 (-0.75, 0.49) | -0.03 (-0.12, 0.05) | -0.1 (-0.74, 0.54) |
| PhenoAge (PC adjusted) | 0.01 (-0.61, 0.62) | -0.07 (-0.16, 0.01) | 0.08 (-0.55, 0.72) |
| GrimAge (PC adjusted) | -0.27 (-0.88, 0.33) | -0.08 (-0.16, 0) | -0.19 (-0.82, 0.44) |
| GrimAge2 (PC adjusted) | -0.07 (-0.66, 0.52) | -0.05 (-0.13, 0.03) | -0.03 (-0.63, 0.58) |
| DunedinPACE | 0.46 (-0.13, 1.05) | -0.05 (-0.13, 0.03) | 0.51 (-0.1, 1.11) |
| **GrimAge DNAm based plasma proteins** |  |  |  |
| PC-DNAm ADM | -0.56 ( -1.16, 0.05) | -0.07 (-0.15, 0.01) | -0.49 (-1.11, 0.14) |
| PC-DNAm B2M | 0.22 (-0.37, 0.82) | -0.07 (-0.15, 0.02) | 0.29 (-0.33, 0.91) |
| PC-DNAm TIMP-1 | -0.54 ( -1.15, 0.06) | -0.08 (-0.16, 0.01) | -0.47 (-1.09, 0.15) |
| PC-DNAm Leptin | -0.53 (-1.15, 0.08) | -0.02 (-0.11, 0.06) | -0.51 (-1.15, 0.12) |
| PC-DNAm CystatinC | 0.39 (-0.23, 1.02) | -0.04 (-0.13, 0.04) | 0.44 (-0.21, 1.08) |
| PC-DNAm PAI-1 | -0.45 (-1.05, 0.16) | -0.06 (-0.14, 0.03) | -0.39 (-1.02, 0.23) |
| PC-DNAm GDF15 | -0.18 (-0.79, 0.43) | -0.07 (-0.16, 0.01) | -0.1 (-0.73, 0.52) |
| Adjustments: DO-HEALTH treatments, age (continuous and categorical >85 years), sex, prior fall, BMI, study site, cancer history, baseline DNAm measure and time with cancer. | | | |

**Supplementary Table 8. Sensitivity analysis of the three-year changes in DNAm measures from baseline in participants with and without incident cancer adjusted for cell counts and lifestyle factors (baseline status of: smoking, education years, number of comorbidities)**

|  | **Adjusted change from BL (95% CI)** | | |
| --- | --- | --- | --- |
| **Biological age measure** | **Cancer case (n = 30)** | **Cancer free (n =747)** | **Difference** |
| **Sensitivity analysis adjusted for cell counts** |  |  |  |
| Horvath (PC adjusted) | -0.17 (-0.76, 0.42) | 0.01 (-0.07, 0.09) | -0.18 (-0.79, 0.43) |
| Hannum (PC adjusted) | 0.04 (-0.54, 0.62) | -0.02 (-0.1, 0.05) | 0.06 (-0.53, 0.66) |
| PhenoAge (PC adjusted) | 0.45 (0.05, 0.85) | -0.02 (-0.08, 0.03) | 0.47 (0.05 , 0.88) |
| GrimAge (PC adjusted) | 0.3 (-0.12, 0.72) | -0.02 (-0.08, 0.04) | 0.32 (-0.11, 0.75) |
| GrimAge2 (PC adjusted) | 0.22 (-0.31, 0.75) | -0.02 (-0.09, 0.05) | 0.24 (-0.3, 0.78) |
| DunedinPACE | 0.67 (0.12, 1.22) | -0.02 (-0.09, 0.06) | 0.69 (0.13, 1.26) |
| **GrimAge DNAm based plasma proteins** |  |  |  |
| PC-DNAm ADM | 0 (-0.39, 0.4) | -0.01 (-0.06, 0.04) | 0.01 (-0.4, 0.42) |
| PC-DNAm B2M | 0.53 (0.05, 1.01) | -0.03 (-0.09, 0.04) | 0.55 (0.06, 1.05) |
| PC-DNAm TIMP-1 | 0.13 (-0.05, 0.31) | 0 (-0.03. 0.02) | 0.13 (-0.05, 0.32) |
| PC-DNAm Leptin | -0.03 (-0.54, 0.49) | 0.01 (-0.06, 0.08) | -0.04 (-0.56, 0.49) |
| PC-DNAm CystatinC | 0.57 (0, 1.14) | -0.03 (-0.1, 0.05) | 0.6 (0, 1.19) |
| PC-DNAm PAI-1 | 0.18 (-0.14, 0.51) | 0 (-0.05, 0.04) | 0.19 (-0.15, 0.52) |
| PC-DNAm GDF15 | 0.39 (0.05, 0.74) | -0.01 (-0.05, 0.04) | 0.4 (0.05, 0.75) |
| **Sensitivity analysis adjusted for smoking, education years and comorbidities** |  |  |  |
| Horvath (PC adjusted) | 0.09 (-0.52, 0.7) | 0.02 (-0.06, 0.1) | 0.07 (-0.56, 0.7) |
| Hannum (PC adjusted) | -0.09 (-0.71, 0.53) | -0.03 (-0.11, 0.06) | -0.06 (-0.71, 0.58) |
| PhenoAge (PC adjusted) | 0.03 (-0.59, 0.65) | -0.08 (-0.17, 0) | 0.11 (-0.53, 0.75) |
| GrimAge (PC adjusted) | -0.29 (-0.9, 0.32) | -0.09 (-0.17, -0.01) | -0.2 (-0.83, 0.43) |
| GrimAge2 (PC adjusted) | -0.04 (-0.63, 0.55) | -0.06 (-0.14, 0.02) | 0.01 (-0.59, 0.62) |
| DunedinPACE | 0.47 (-0.13, 1.06) | -0.06 (-0.14, 0.02) | 0.52 (-0.09, 1.13) |
| **GrimAge DNAm based plasma proteins** |  |  |  |
| PC-DNAm ADM | -0.57 (-1.17, 0.04) | -0.08 (-0.17, 0) | -0.48 (-1.11, 0.14) |
| PC-DNAm B2M | 0.24 (-0.37, 0.84) | -0.07 (-0.15, 0.01) | 0.31 (-0.31, 0.93) |
| PC-DNAm TIMP-1 | -0.57 (-1.17, 0.04) | -0.08 (-0.17, 0) | -0.48 (-1.11, 0.14) |
| PC-DNAm Leptin | -0.57 (-1.19, 0.05) | -0.03 (-0.12, 0.05) | -0.54 (-1.17, 0.1) |
| PC-DNAm CystatinC | 0.41 (-0.22, 1.03) | -0.04 (-0.13, 0.04) | 0.45 (-0.19, 1.1) |
| PC-DNAm PAI-1 | -0.45 (-1.06, 0.16) | -0.06 (-0.15, 0.02) | -0.38 (-1.01, 0.24) |
| PC-DNAm GDF15 | -0.17 (-0.78, 0.44) | -0.08 (-0.17, 0) | -0.09 (-0.72, 0.54) |
| Adjustments: DO-HEALTH treatments, age (continuous and categorical >85 years), sex, prior fall, BMI, study site, cancer history, baseline DNAm measure and time with cancer, baseline cell counts (NK, BAS, MONO, EOS, Bcells, CD4, CD8) and change from baseline. EAA is shown in standardized units. | | | |
